# Speckle-based curvature optical metrology

**DOI:** 10.1038/s41377-026-02257-x

**Published:** 2026-04-08

**Authors:** Hongchang Wang, Riley Shurvinton, Paresh Pradhan, Kawal Sawhney

**Affiliations:** Diamond Light Source Ltd, Harwell Science and Innovation Campus, Didcot, OX11 0DE UK

**Keywords:** Adaptive optics, X-rays

## Abstract

Advanced metrology methods are continually being developed and refined to meet the demanding quality standards of high-performance X-ray mirrors. Among these, interferometric techniques are the most widely used for measuring the height maps of optical surfaces. However, they often encounter limitations when applied to strongly curved or freeform surfaces, primarily due to high fringe density caused by steep slope. To address these challenges, we have developed a laser Speckle-based Curvature Optical Metrology instrument (SCOM) for measuring the two-dimensional surface curvature maps. This technique offers an alternative for characterizing complex optical surfaces by using a digital image correlation algorithm to extract curvature information from the speckle pattern, which effectively acts as a set of wavefront markers. We have demonstrated the effectiveness of this method for measuring strongly curved mirrors with a radius of curvature from 10 m down to 100 mm. Additionally, we have applied this technique to online deterministic figuring of optical surfaces, in-situ stress measurements during multilayer deposition processes, and the characterization of deformable mirrors. This technique shows great promise not only for high precision metrology of X-ray mirrors used in synchrotron radiation facilities, free-electron lasers, and space and astronomical observatories, but also for freeform optical components in advanced industrial applications.

## Abstract (web summary)

Speckle-based Curvature Optical Metrology (SCOM) instrument enables high-precision two-dimensional curvature mapping of strongly curved and freeform optical surfaces, supporting deterministic figuring, in-situ stress monitoring, and advanced X-ray mirror characterization.

## Graphical abstract

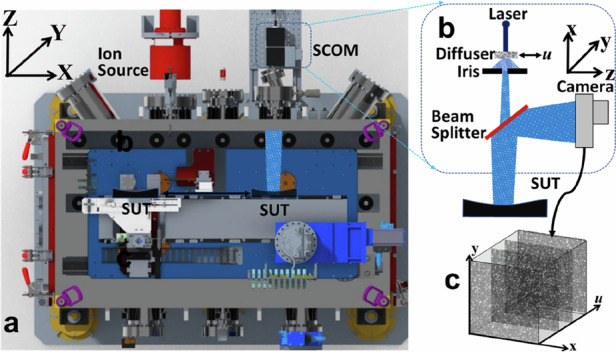

## Introduction

The high-precision metrology of mirrors plays a crucial role in advancing the performance and capabilities of modern X-ray optics, particularly in scientific applications such as synchrotron radiation, X-ray astronomy, and X-ray microscopy.^1^ For instance, to exploit optimally modern high-brilliance X-ray sources, experiments increasingly require high accuracy reflective optics with peak-to-valley figure errors of the order of 1 nm over spatial periods from 0.1 mm up to tens of centimetres.^2^ This is particularly the case for X-ray instrumentation seeking to exploit the spatial coherence of the X-ray beams. Correction of figure errors at these scales is possible using deterministic figuring techniques such as elastic emission machining, computer controlled robotic polishing, differential coating and ion beam figuring.^3–7^ Nevertheless, all figure correction methods depend on accurate metrology information to determine the necessary corrections to the surface topography.^8–11^

Coordinate Measuring Machines (CMMs) use mechanical or optical probes to map surface geometry in three-dimensional (3D) space to provide high geometric accuracy over large areas with flexible probe types, but this approach suffers from low speed and throughput.^12^ Interferometric metrology is an instantaneous method for the measuring the two-dimensional (2D) height map of the surface under test (SUT) relative to the Fizeau’s transmission reference optic. Fizeau interferometry (FI) is suited for planar and spherical surfaces, depending on the transmission optic which is used.^13,14^ However, dedicated and careful calibration of the optical system errors from the reference optics and, if applicable, a Computer-Generated Hologram (CGH) is required.^15,16^

The field-of-view (FOV) of conventional interferometric systems often limits the ability to fully capture the surface of large mirrors. To overcome this limitation, Fizeau- and micro-interferometer stitching techniques are increasingly being developed to extend the measurement capability of interferometers to large apertures while maintaining high resolution.^17–22^ This technique involves taking multiple interferometric measurements across different sub-apertures of the mirror surface and then using computational algorithms to merge the data into a single, high-resolution surface map. A complex stitching process involves measuring overlapping regions of the surface using sub-aperture interferometers and applying digital alignment techniques to correctly align the sub-apertures by minimizing the systematic errors such as piston, tilt, and lateral offsets. However, their implementation for both shorter radius and strongly aspheric surfaces is non-trivial, which has led to the development of so-called relative angle determinable stitching interferometry (RADSI).^23^ This approach improves performance of stitching interferometry for strongly-curved mirrors, but requires a reference mirror during the measurement and employs advanced algorithms and significant computation to accurately determine relative angles and stitch data. Furthermore, digital holographic microscopy (DHM) is a fast, non-invasive interferometric technique with high phase-measurement accuracy, enhanced recently by deep learning, and widely used for surface-profile measurements.^24–26^ The main challenge in current DHM is that traditional aberration-compensation methods are inadequate for large-aperture measurements, making precise, efficient characterization of high-Numerical Aperture (NA) objects difficult.^27^

Nanometer Optical Component Measuring Machines (NOM) and Long Trace Profilers (LTP) are widely used in X-ray metrology for measuring moderately curved mirrors, and provide 1D slope profiles with lateral resolution limited to 1–2 mm due to the pencil beam size used in scanning.^28–32^ Although the NOM can measure the full surface by combining with sagittal and diagonal traces, this is a time-consuming process, and the extended duration requires a stringent measurement environment and minimal instrumental drifts. Recently, a Speckle Angular Measurement (SAM) approach has been developed to retrieve both slope and curvature information by utilising an advanced sub-pixel tracking algorithm for the speckle images.^33^ While 2D slope and curvature maps can be measured by analyzing subset speckle images, the sagittal FOV is limited to just a few millimetres due to the smaller camera. Additionally, an autocollimator must be used to correct for pitch errors in the carriage slide.

Consequently, there is significant interest to develop a metrology system which accounts for large mirror apertures, complex geometries, and steep surface curvatures. In this paper, we have developed a Speckle-based Curvature Optical Metrology (SCOM) instrument by implementing a large camera for measuring two-dimensional surface curvature maps. To address the limitations of restricted FOV, a stitching technique has been used to merge the data into a single surface curvature map. Importantly, the diffuser was mounted on a high precision linear stage, and multiple speckle images have been collected to significantly improve the measurement precision. In addition, the instrument is highly compact and portable, making it well-suited for in-situ, on-machine measurement applications.^34^ We have demonstrated the use of this technique for the deterministic figuring of X-ray mirrors, in-situ stress measurement during multilayer deposition process and metrology of freeform mirrors.

**Fig. 1:**
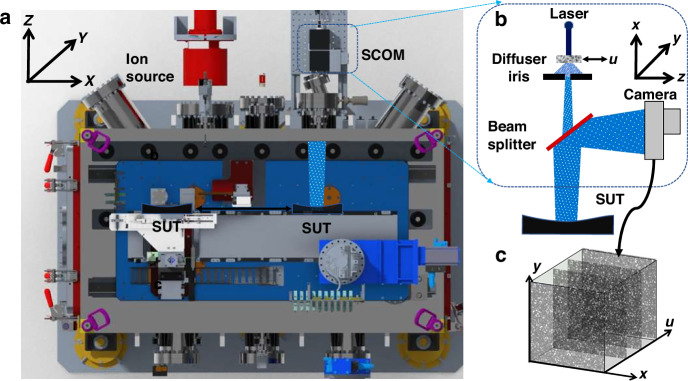
Schematic representation of the experimental setup for SCOM, which has been installed on an IBF system. **a** The top view of a 3D model of the system, where SUT can be translated between ion source and SCOM, and the curvature measurement can be carried out before and after the IBF process. The SCOM is mounted on a fixed platform and detected the speckled signal from one angled viewport once the SUT has been transferred to SCOM position. **b** The optical schematic representation of the SCOM setup for 2D surface curvature measurement of a SUT. **c** The acquired stack of speckle images in Cartesian coordinates

As one application example, the first SCOM instrument is retrofitted on an existing Ion Beam Figuring (IBF) system, which is used for the final shaping and deterministic figuring of high-precision optical surfaces.^35^ A schematic representation of the IBF system and SCOM is presented in Fig. 1. The SUT has been mounted on a motorized four-axis motion stage (X, Y, Z, and θy), which can be used to translate the SUT between the ion source and the SCOM before and after IBF process. The SCOM is composed of a collimating laser, a diffuser mounted on a linear stage, an iris, a beam splitter and a camera with a large FOV. To improve the stability and minimize the power dissipation, a low-power laser diode (4.5 mW at 405 nm) was used. The collimated light was shone on a ground glass diffuser to generate the speckle pattern. The diffuser was mounted on a linear translation stage to enable multiple speckle images to be collected at different diffuser positions. Both the spatial resolution and signal-to-noise ratio can be improved by scanning and averaging multiple speckle images.

To further improve the spatial resolution of the SUT, an iris has been installed downstream of the diffuser to effectively reduce the illumination area of speckle patterns. The mean speckle size (${{\rm{s}}}_{{\rm{s}}}$) at the camera plane is given by ${{\rm{s}}}_{{\rm{s}}}={\rm{\lambda }}{\rm{L}}/{\rm{D}}$, where ${\rm{\lambda }}$ is the wavelength of light, ${\rm{D}}$ is the aperture of the iris, and ${\rm{L}}$ is the distance traveled by the laser beam from the iris to the camera via the SUT and beam splitter. As expected, the speckle size decreases with increasing iris aperture but increases with the distance between the SCOM and the SUT. The spatial resolution is limited by the large illumination area on the diffuser, as the mirror surface information is convolved with the speckle pattern. While closing the iris can improve resolution, it increases the speckle size, limiting the speckle window size. Therefore, it is essential to optimize iris size to improve the spatial resolution.

After the diffuser, the laser beam passes through the beam-splitter (50:50) so that part of it projects onto the mirror surface. The beam path is sealed with a special designed cage to minimize the air turbulence. The beam reflected by the SUT, still carrying the speckle pattern, is recorded by a high-resolution camera with a large FOV. A VIEWWORKS VC-151MC camera, equipped with a Sony IMX411 CMOS sensor (active area: 53 mm × 40 mm), was used to capture a large area of the speckle pattern. The camera features a resolution of 14192 (H) ×10640 (V) pixels with a 3.76 μm pixel size. To reduce raw image size and processing time, 12 × 12 pixel binning was applied to all examples presented in this paper. To prevent interference between the reflected beam from the standard viewport and the beam from the SUT, a custom angled viewport was installed in front of the SCOM on the high vacuum IBF chamber. Data acquisition at a single position with 51 images in each stack takes around ten minutes. Using a 5 mm coarse step size between positions, a 100mm-long mirror measured at 21 positions along the length can be measured in around three and a half hours.

The description of speckle tracking relies on the two coordinate systems illustrated in Fig. 1. The coordinates $\left(X,Y\right)$ correspond to the tangential (length) and sagittal (width) directions of the SUT, respectively. In addition, the coordinates $\left(x,y\right)$ represent the horizontal and vertical axes of the camera image plane, respectively. It is important to note that, due to the divergence of the speckle beam, a spatial scaling factor, denoted as ς, exists between the two coordinate systems.

1 $$\left\{\begin{array}{c}X=\varsigma x=\frac{P}{p}x\\ Y=\varsigma y=\frac{P}{p}y\end{array}\right.$$

Here $p$ and $P$ denotes the camera physical and effective pixel size, respectively. This effective pixel size $P$ can be determined by tracking a reference feature on the mirror in $x,y$ as a function of its translation distance $X,Y$, described further in the Materials and Methods section (Fig. 8).

**Fig. 2:**
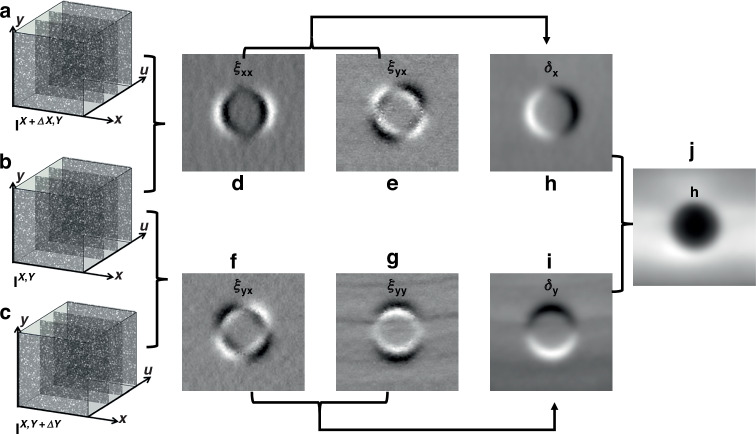
A schematic illustration of extraction of speckle displacement (curvature), slope and height images of a test mirror. **a**–**c** The acquired stack of speckle images in Cartesian coordinates at various X and Y positions. **d**–**g** The extracted speckle displacement along horizontal, diagonal and vertical direction. (**h**, **i**) The constructed surface slope along horizontal (x) and vertical (y) direction, respectively. **j** The integrated surface height map from the slope images (**h**, **i**)

Figure 2 presents a schematic of the extraction process for speckle displacement (curvature), slope, and height maps of a SUT. A circular beam removal function (BRF), defined by a 5 mm diameter aperture, was etched onto the SUT using the ion beam over 300 s.^36^ With the SCOM instrument facing to the SUT, a stack of speckle images ${I}^{X,Y}(x,y,u)$ at position ($X,Y$) was collected by translating the diffuser along $u$ across multiple positions. The stack of speckle image can be written as ${I}^{X,Y}$ by omitting $\left(x,y,u\right)$ for clarity. The SUT was then moved to position ($X+\Delta X,Y$) by using the scanning carriage with a small step size $\Delta {\rm{X}}$, here chosen to be 0.25 mm. As a result, the beam was projected onto different regions of the SUT, and a second stack of speckle images ${I}^{X+\Delta X,Y}$ are acquired.

In this process, the speckle pattern serves as a carrier of the SUT surface information. The speckle pattern recorded by the camera undergoes a shift corresponding to variations in the curvature of the SUT. Speckle displacements in both the $x$ direction and $y$ direction are extracted from Pearson correlation coefficient generated from the speckle stacks ${I}^{X,Y}$ and ${I}^{X+\Delta X,Y}$,^37–40^ and a pixel wise data analysis was used by applying a sub-pixel image registration algorithm to generate the displacement maps ${\xi }_{{xx}}$ and ${\xi }_{{xy}}$ (omitting $\left(x,y\right)$ for clarity).^33,41^ Similarly, by translating the SUT to position ($X,Y+\Delta Y$) with step size $\Delta {\rm{Y}}$, the displacements maps ${\xi }_{{yx}}$ and ${\xi }_{{yy}}$ are recovered by comparing the image stacks ${I}^{X,Y}$ and ${I}^{X,Y+\triangle Y}$.

From these displacement maps, the curvature (defined as the second derivative of height) of the surface can be obtained.^42^ The curvature $\kappa$ is often expressed as the inverse of the radius of curvature $R$, and it can then be expressed in terms of the derived speckle displacement maps as:

2 $$\left\{\begin{array}{c}{{\rm{\kappa }}}_{{xx}}=\frac{1}{{R}_{{xx}}}=\frac{{d}^{2}h}{d{x}^{2}}=\frac{d{\delta }_{x}}{{dx}}=\frac{p{\xi }_{{xx}}}{2d\varDelta X}\\ {{\rm{\kappa }}}_{{xy}}=\frac{1}{{R}_{{xy}}}=\frac{{d}^{2}h}{{dxdy}}=\frac{d{\delta }_{x}}{{dy}}=\frac{{p\xi }_{{xy}}}{2d\varDelta X}\\ {{\rm{\kappa }}}_{{yx}}=\frac{1}{{R}_{{yx}}}=\frac{{d}^{2}h}{{dydx}}=\frac{d{\delta }_{y}}{{dx}}=\frac{{p\xi }_{{yx}}}{2d\varDelta Y}\\ {{\rm{\kappa }}}_{{yy}}=\frac{1}{{R}_{{yy}}}=\frac{{d}^{2}h}{d{y}^{2}}=\frac{d{\delta }_{y}}{{dy}}=\frac{p{\xi }_{{yy}}}{2d\varDelta Y}\end{array}\right.$$

Here, $h$ and $\delta$ denote the height and slope of the surface, respectively, while $d$ represents the distance travelled by the laser beam from the SUT to the camera. To accurately determine the distance $d$, one can retrieve the angular scaling factor (denoted as $\tau$)^33^, defined as:

3 $$\tau =\frac{p}{2d}$$

This can be retrieved by tracking the speckle displacement as a function of the mirror rotation angle, which is described further in the materials and methods section. Increasing $d$ and reducing $p$ can improve the signal-to-noise ratio in speckle-tracking displacement measurements; however, increasing $d$ enlarges speckle size, and reducing $p$ reduces the field of view. Therefore, a trade-off is required among distance and detector pixel size to optimize the field of view and angular sensitivity.

The mean curvature, or equivalently the mean inverse of the radius of curvature, can be calculated from the curvature along the x and y directions using the following equation:

4 $$\kappa =\frac{1}{R}=\frac{{{\kappa }}_{{xx}}+{{\kappa }}_{{yy}}}{2}$$

As shown in Fig. 2d–g, the speckle displacement maps are proportional to the curvature maps along the horizontal, vertical, and two diagonal directions, respectively. Once the curvature maps are obtained, the slope maps ${\delta }_{x}$ and ${\delta }_{y}$ can be computed by integrating the curvature map with the Fourier transform integration method^43,44^, using the following expression:

5 $$\left\{\begin{array}{l}{\delta }_{x}(x,y)=\frac{{pP}}{2d\varDelta X}{{\mathcal{F}}}^{-1}\left[\frac{{\mathcal{F}}\left[{\xi }_{{xx}}(x,y)+i{\xi }_{{xy}}(x,y)\right](m,n)}{2\pi i(m+{in})}\right](x,y)\\ {\delta }_{y}\left(x,y\right)=\frac{{pP}}{2d\varDelta Y}{{\mathcal{F}}}^{-1}\left[\frac{{\mathcal{F}}\left[{\xi }_{{yx}}\left(x,y\right)+i{\xi }_{{yy}}\left(x,y\right)\right]\left(m,n\right)}{2\pi i\left(m+{in}\right)}\right]\left(x,y\right)\end{array}\right.$$

where ${{\mathcal{F}}}^{-1}$ (${\mathcal{F}}$) is the inverse (forward) Fourier operations and $\left(m,n\right)$ represent the reciprocal space coordinates corresponding to $\left(x,y\right)$.

Figure 2h, i shows the integrated horizontal and vertical slope maps for the BRF. For the horizontal slope, the slope varies left-to-right, with a dark-to-bright transition indicating increasing slope from negative to positive in the x-direction. The vertical slope varies bottom-to-top, showing a similar transition but in the y-direction. Finally, the height map (Fig. 2j) can be derived by integrating the slope maps using the following equation:

6 $$h(x,y)=P{{\mathcal{F}}}^{-1}\left[\frac{{\mathcal{F}}\left[{\delta }_{x}(x,y)+i{\delta }_{y}(x,y)\right](m,n)}{2\pi i(m+{in})}\right](x,y)$$

The height map for the BRF shown in Fig. 2j has a circular, dome-like shape, with a valley at the centre, and the smooth and symmetric grayscale gradient confirms the spherical geometry etched by the ion source.

## Results

On-machine metrology enables online monitoring of surface shape evolution during the IBF process, allowing for more accurate and efficient corrections. To demonstrate the application of SCOM for online metrology the IBF process, as illustrated in Supplementary Information Figure S1, the curvature of a test mirror was measured before and after etching with the ion beam. These maps reveal circular tooling marks on mirror surface, and the curvature maps are quite distinctive along different orientations. Using the ion beam, three cross marks were etched for 60 s, 90 s, and 120 s at a fixed Z-position of 55 mm, while circular beam removal functions (BRFs) 5 mm in diameter were etched at varying sample-to-ion source distances with Z set to 55 mm, 50 mm, and 45 mm. It should be noted that the curvature measurement with SCOM were carried out without taking the mirror out of the IBF chamber.

**Fig. 3:**
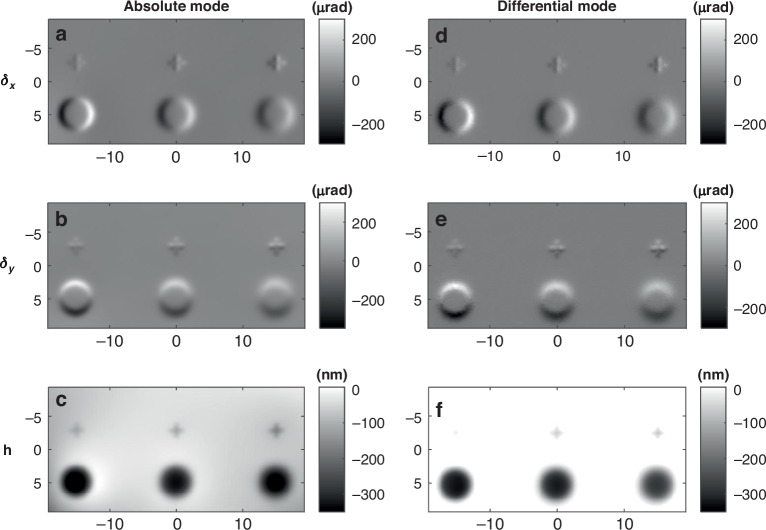
Retrieved slope and height maps for a test mirror under absolute and differential processing modes. **a–c** Horizontal slope, vertical slope, and height maps of the test mirror processed in *absolute mode*. **d–f** Corresponding maps obtained under *differential mode* processing

The previously described working mode for the SCOM is called the absolute mode, and it measures the curvature of the SUT from a single stack of images. In contrast, the SCOM can also be operated in differential mode, which directly compares two image stacks before and after etching (${{\rm{I}}}_{1}^{{\rm{X}},{\rm{Y}}}$ and ${{\rm{I}}}_{2}^{{\rm{X}},{\rm{Y}}}$, respectively). Changes in the slope from etching induce shifts in the speckle pattern, allowing direct retrieval of the differential slope map before and after etching. Figure 3 provides a comprehensive comparison of the retrieved slope and height maps after ion beam etching, obtained using the absolute and differential modes. Figure 3a–c show the horizontal slope, vertical slope, and surface height maps obtained using the absolute mode, and Fig. 3d–f present the corresponding maps for the same mirror using the differential mode. For slope maps, the absolute mode provides smoother and higher-quality data—especially at the BRF edges—as they are derived from curvature. In contrast, the differential mode produces noisier slope maps but yields etching rate estimates that align more closely with FI (Zygo Verifire HDX) measurements.

A super-Gaussian function was used to fit all BRF profiles. For instance, at Z = 45 mm, the etching rates are 1.016 nm/s (absolute), 1.031 nm/s (differential), and 1.057 nm/s (FI); at Z = 55 mm, they are 1.184 nm/s (absolute), 1.051 nm/s (differential), and 1.165 nm/s (FI), confirming increased etching rates when the sample is closer to the ion source. While both modes show good overall agreement, low-frequency errors are more evident in the height maps from the absolute mode due to noise accumulation during the two-step integration (curvature → slope → height). In contrast, the differential mode involves only a single integration (slope → height), resulting in reduced low-frequency drift. However, the differential mode poses practical challenges: it requires precise repositioning of the sample for consecutive scans, and its data processing is more time-consuming due to larger speckle displacements. The results confirm that the SCOM system can reliably capture curvature changes across different orientations, offering valuable insights into how the ion beam etching rate varies with etching parameters. The ability to retrieve accurate slope and curvature maps using both processing modes highlights the versatility of the SCOM system, enabling its adaptation to various applications depending on specific metrology requirements, and its particular value for on-machine metrology for mirror polishing, shaping, or coating deposition.

**Fig. 4:**
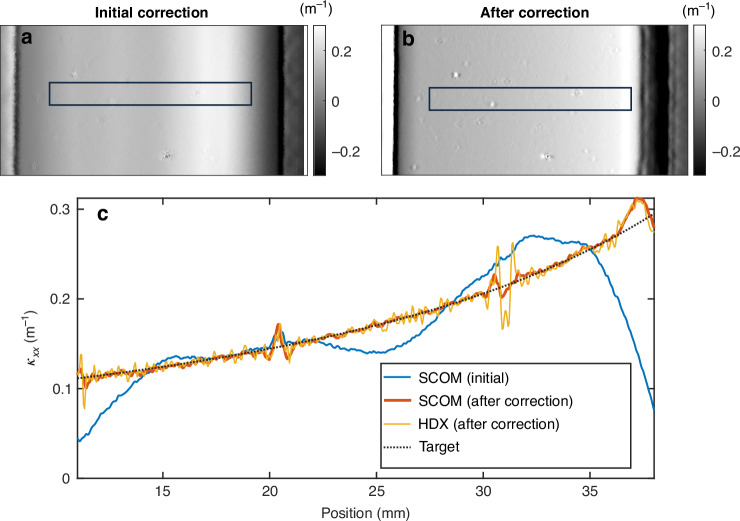
Measured curvature maps with SCOM for a strongly curved ellipse mirror during IBF process. **a**, **b** The curvature maps along the tangential direction of the mirror are shown for initial and final IBF correction iterations. The scale bar is 5 mm. **c** Line profile extracted from the highlighted region of the curvature map, showing the convergence of the curvature to the target ellipse. The extracted curvature from a FI measurement is also shown for comparison, giving excellent agreement with the SCOM

To further demonstrate the capability of the proposed SCOM system for online metrology in IBF, we conducted an experiment involving a strongly curved elliptical mirror measured in situ during the etching process. The target figure for the mirror was a tangential elliptical surface,^45^ with the source and image focal points located at distances of p = 45 m and q = 0.043 m, respectively. The grazing angle of incidence at the centre of the mirror was θ = 0.825°. This configuration presents a significant metrology challenge due to the steep curvature and tight alignment tolerances, with a sag of around 18 µm over the 27 mm-wide active area. As illustrated in Fig. 4a, b, the curvature maps along the tangential direction of the mirror are shown for two IBF correction iterations. A representative line profile extracted from the highlighted region of the curvature map is shown in Fig. 4c, offering a clearer view of the surface evolution. During the first correction, the curvature profile deviated significantly from the target shape, indicating substantial low-frequency errors. After further correction by multiple iterations of IBF, the curvature was significantly improved, bringing it very close to the target elliptical figure. The curvature was also compared with a measurement obtained using stitching FI, with an 0.45 mm moving average window applied to smooth out high-frequency noise. The SCOM is shown to give excellent agreement with the FI measurement.

It is important to note that such strongly curved mirrors are extremely difficult to measure using conventional interferometric systems. In this example, the FI measurements took 6.5 h for a single scan, due to the small acquisition window and step size required to obtain accurate data for such a strongly curved mirror. However, SCOM successfully retrieved detailed curvature maps in one hour for this case, enabling precise monitoring and control of the figuring process. Overall, this example highlights the significant advantage of using SCOM for online metrology in IBF. By providing immediate curvature feedback during processing, SCOM reduces the need for iterative cycles of figuring and offline measurements, which are often time-consuming and resource-intensive.

To demonstrate the applicability of the proposed technique for characterizing strongly curved mirrors, a second SCOM system was developed and integrated into a dedicated gantry platform. The entire setup is housed within a thermally isolated enclosure to minimize environmental disturbances. In this configuration, the SCOM instrument is mounted on an air-bearing linear translation stage, providing smooth and accurate scanning motion. The SUT is positioned face-up on a hexapod stage, which enables precise control of both translational and angular alignment through six degrees of freedom. This setup allows for optimal positioning of the mirror relative to the SCOM optical axis and facilitates accurate measurements across a wide range of mirror geometries.

**Fig. 5:**
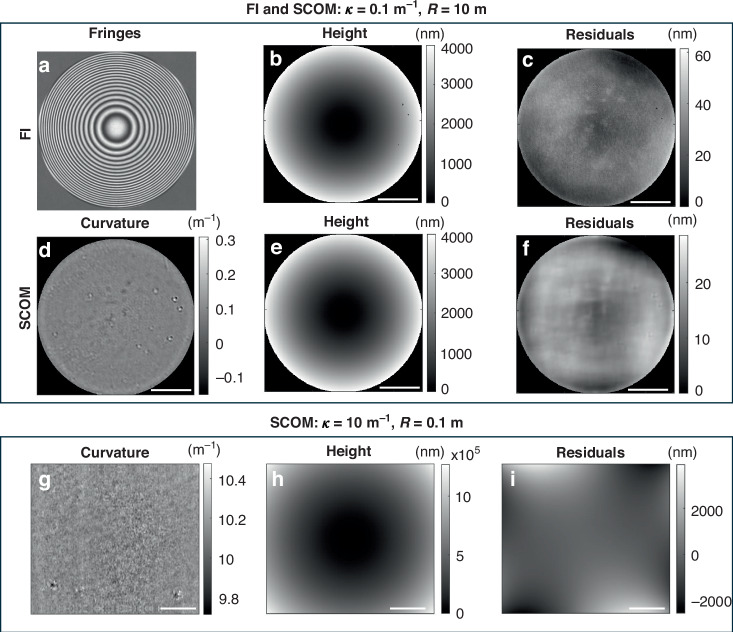
Measured curvature and height maps for two strongly curved mirrors using an interferometer and SCOM. **a** Raw interference fringe pattern and **b** measured height map using an interferometer (FI) for a mirror with a curvature of 0.1 m⁻¹. **c** The residual height error versus the best-fit sphere. **d** Retrieved curvature, **e** height map, and **f** residual height error using SCOM for the same mirror, demonstrating good agreement with the FI. **g** Retrieved curvature, **h** height map, and **i** residual height error using SCOM for a strongly curved mirror with a curvature of 10.0 m⁻¹. The scale bar is 5 mm for all plots

As shown in Fig. 5, two spherical mirrors with specified mean curvatures of $\kappa =0.1{{\rm{m}}}^{-1}(R=10{\rm{m}})$ and $\kappa =10.0{{\rm{m}}}^{-1}(R=100{\rm{mm}})$ were measured using SCOM. To validate SCOM’s accuracy, the first spherical mirror with a curvature of 0.1 m⁻¹ was also measured using the FI. The raw interference fringe is shown in Fig. 5a, with the corresponding height map in Fig. 5b, yielding a curvature of 0.086 m^−1^ and 0.085 m^−1^ along horizontal and vertical direction, respectively. As shown in Fig. 5e, the height map for SCOM is nearly identical to the one measured with FI in Fig. 5b. Notably, The SCOM-retrieved curvature map (Fig. 5d) shows a mean curvature of 0.085 ± 0.012 m^−1^, in excellent agreement with the FI result. The sphere fitting has been applied to the height map, and the corresponding residual height error for FI and SCOM are shown in Fig. 5c, f), respectively. The standard deviation between the FI and SCOM height error is about 5.1 nm, and the major error contribution for SCOM is due to the integration error. Surface artifacts are more clearly visible in the curvature map than in the height profile. The FI residual height, SCOM curvature, and SCOM residual height all show good qualitative agreement for the measured surface artefacts, with repeated features clearly visible between all three maps.

The mirror with 10.0 m⁻¹ curvature could not be measured using FI, due to the steep gradients. However, it was successfully measured using the SCOM, with the curvature and height maps shown in Fig. 5g and Fig. 5h, respectively. As shown in Fig. 5h, the peak-to-valley value of the height map is approximately 1 mm over a 20 mm span, which is very challenging to measure precisely using many existing metrology instruments. The measured mean curvature was 10.13 ± 0.05 m⁻¹, closely matching the specified value. The curvature map also reveals non-uniformities introduced during the polishing process. The residual height error for this mirror versus the best-fit sphere is shown in Fig. 5i, and the large peak-to-valley of the height error shows the poor quality of this mirror.

**Fig. 6:**
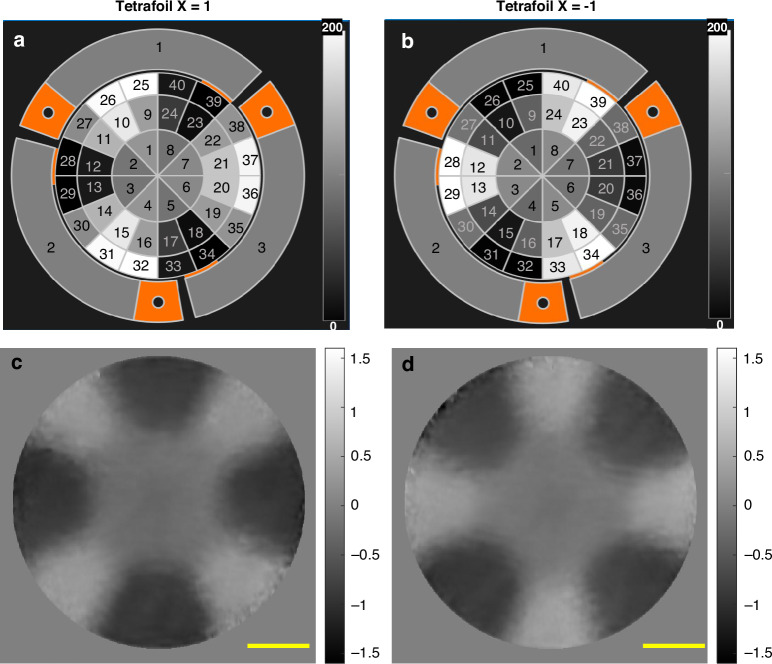
Voltage and mean curvature map for a deformable mirror using Zernike Tetrafoil modes. **a** The voltage map of the deformable mirror (**a**) Tetrafoil X *=* 1 (**b**) Tetrafoil X *=* -1. Retrieved mean curvature with (**c**) Tetrafoil X *=* 1 (**d**) Tetrafoil X *=* -1, and the scale bar unit is m^-1^. The scale bar is 2 mm

To demonstrate the practical application of the proposed SCOM system for the precise characterization of freeform mirrors, we conducted a series of experiments on a piezoelectric deformable mirror. The mirror features a circular pupil with a diameter of 10 mm and is suspended by biomorph benders, which allow for controlled deformation in response to applied voltages. The mirror is equipped with 40 individually addressable actuators, each capable of receiving voltages ranging from 0 V to 200 V, enabling a wide range of surface shape modulations. In this study, we employed a pre-defined Zernike polynomial pattern corresponding to the tetrafoil X mode to generate specific surface deformations. The tetrafoil mode was varied from +1 to −1, and the resulting voltage distribution maps applied to the actuators are shown in Fig. 6a, b. The corresponding mean curvature maps derived from the SCOM measurements are presented in Fig. 6c, d, respectively. These maps clearly show that when the tetrafoil X value was changed from +1 to −1, the sign of the mean curvature map was inverted accordingly. This confirms the system’s sensitivity to both the magnitude and direction of surface curvature changes. As anticipated, an increase in applied voltage deformed the mirror into a convex shape, producing a surface with negative curvature. Conversely, decreasing the voltage reversed the deformation, resulting in a concave shape with positive curvature. The measured mean curvature values ranged from −1.5 m⁻¹ to +1.5 m⁻¹, with distinct and sharp transitions observable across the mirror surface. These sharp gradients demonstrate the ability of the SCOM system to capture detailed curvature variations with moderate spatial resolution. Overall, the results demonstrate the potential of the SCOM for accurate, non-contact characterization of freeform optical components. The system is particularly well-suited for mirrors exhibiting complex, non-symmetric shapes that are difficult to measure using conventional metrology instruments.

A third SCOM system has been developed for stress measurements of coated films and mounted on an angled viewport of the newly developed Multilayer Deposition System (MDS).^46^ The MDS utilizes direct current magnetron sputter deposition for monolayer or multilayer fabrication and accommodates a total of 8 cathodes along the carrier’s motion direction. Each cathode, measuring 254 mm × 89 mm, is designed to ensure optimal flux uniformity. For comparison, a Multi-beam Optical Sensor (MOS) from k-Space associated is installed on a separate angled viewport of the same system. The MOS uses a single laser to produce a 2D array of spots reflected from the sample surface onto a high-resolution detector. On flat surfaces, spot spacing remains unchanged, while surface curvature causes spacing variations due to beam deflection. By analysing spacing in two orthogonal directions, the MOS provides curvature data along both horizontal and vertical direction. The curvature of the substrate can be measured with both SCOM and MOS before (κ_1_) and after (κ_2)_ coating process.

The change in curvature (Δκ = κ_1_ – κ_2_) is related to the film stress (σ) by the Stoney equation:

7 $$\sigma =\frac{{M}_{s}{h}_{s}^{2}}{{6(1-{\nu }_{s})h}_{f}}\Delta \kappa$$

The other parameters in this equation are the film thickness (h_f_), biaxial Young’s modulus of the substrate (M_s_) Poisson’s ratio of the substrate (ν_s_), and the substrate thickness (h_s_).

**Fig. 7:**
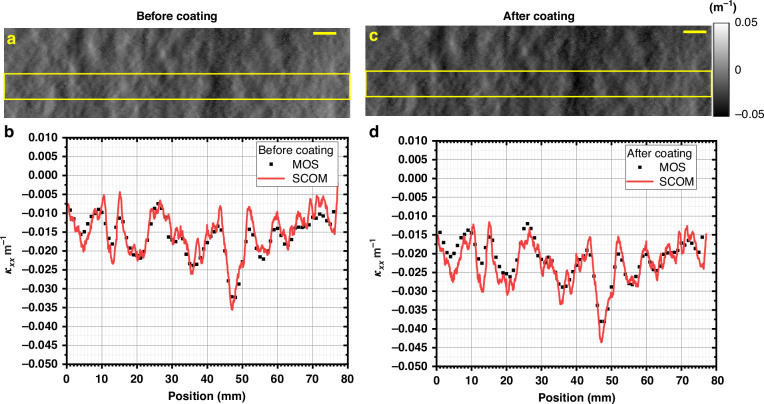
Comparison of curvature map and line profiles between MOS and SCOM before and after coating. **a** Curvature map retrieved from SCOM (**b**) and corresponding line profile between MOS and SCOM before coating. **c** Corresponding map and (**d**) line profile after coating. The yellow box indicates the region where the line profile was extracted. The scale bar is 5 mm

Figure 7 provides a detailed comparison between the curvature measurements obtained using the SCOM and the MOS before and after the coating process. In Fig. 7 a, the curvature map retrieved from SCOM prior to coating is shown, capturing the non-uniform surface curvature distribution across the test mirror. Figure 7b presents the corresponding line profile comparison between the SCOM (red line) and MOS (black squares) systems, extracted along the region marked by the yellow box. The agreement between the two profiles before coating indicates strong consistency and reliability of the SCOM measurements.

The W/B_4_C multilayer with periodic thickness of 3.52 nm and number of period *N* = 100 has been deposited, and the deposition power of W and B_4_C were 100 W and 400 W respectively. The optimized deposition rates of W and B_4_C are 0.1368 nm/s and 0.1748 nm/s, respectively. The multilayers were deposited at a fixed working gas pressure of 3.1 × 10^−3^ mbar. After the coating was applied, the surface was remeasured using both systems. Figure 7c shows the updated curvature map retrieved by SCOM post-coating, revealing the influence of the coating process on the mirror’s surface curvature. Figure 7d presents the line profile comparison after coating, again extracted from the same marked region. Notably, the comparison allows for direct observation of any curvature deviations introduced by the coating layer and serves to validate the SCOM system’s sensitivity to such changes. The average curvature changes for MOS and SCOM are −0.0054 m⁻¹ and −0.0076 m⁻¹, corresponding to stresses of −178 MPa and −250 MPa, respectively. These results indicate that the W/B₄C multilayer induces compressive stress in the silicon substrate. This difference is primarily attributed to the disparity in spatial resolution: the MOS measurements are averaged over a 3 mm × 3 mm region, whereas the SCOM data are processed over a much finer region of 0.2 mm × 0.2 mm. Overall, this measurement highlights the effectiveness and consistency of SCOM for detecting the stress changes due to the coating process, and demonstrates another application of the SCOM to on-machine metrology.

## Discussion

In summary, we have developed a novel curvature optical metrology system, which is designed to address the challenges associated with measuring large-aperture mirrors, complex geometries, and steep surface curvatures. SCOM can produce 2D curvature information, which can then be used for deriving slope and height profiles accordingly. The mirror curvature metrology instrument is versatile and compact, and it can be installed on an existing mirror fabrication instrument or metrology gantry. We have demonstrated that it can be used to measure strongly curved mirrors with a radius of curvature down to 100 mm, which are challenging for existing interferometers using flat transmission optics. In addition to the larger radius range and moderate spatial resolution, high precision can be further achieved by collecting the speckle images at multiple diffuser positions. The calibration procedures for both angular scaling factor and spatial scaling factor have been proposed. In addition, a two-dimensional surface curvature map for larger mirrors can be produced by collecting speckle data from overlapped sub-apertures and stitching retrieved curvature data together. The system has been successfully applied in various advanced optical applications, including deterministic figuring of X-ray mirrors, in-situ stress monitoring during multilayer deposition, and the metrology of freeform optical components. The application of SCOM as an online metrology tool not only improves efficiency but also enhances precision in the fabrication of freeform and strongly curved optical surfaces. Furthermore, with smaller subset window sizes and iris aperture sizes, better spatial resolution below 0.2 mm is achievable. Such flexibility will enable speckle-based instruments to perform metrology on mirror surfaces within the most desirable mid-spatial frequency range, which is crucial for simulating the X-ray beam performance^47^. Importantly, complex stitching algorithms can be avoided for stitching the curvature data, since any parasitic errors affecting the motion only contribute to piston errors in curvature measurements.

To place the SCOM instrument in context with other metrology techniques, Table 1 summarizes the key specifications of several mirror-surface metrology techniques, including resolution, uncertainty, measurement geometry and portability. FI and RADSI deliver the highest accuracy, achieving sub-nanometre height repeatability and fine lateral resolution, but they require long acquisition sequences and are generally less portable. RADSI further depends on extremely precise angular alignment between the surface under test and the reference flat, which makes the system more sensitive to vibration and temperature fluctuations. CGH-based interferometry can reach high precision across a wide range of curvatures, but it requires a custom CGH for different optic, and stitching CGHs for long mirrors adds processing complexity. NOM/LTP instruments provide robust long-trace measurements with excellent repeatability but lower lateral resolution and are limited to 1D profiles, making them best suited for flat or weakly curved optics. Although the SAM instrument attains high-precision pseudo-2D measurements through a 1D scanning method, its overall field of view remains restricted by the small camera size. In contrast, SCOM provides moderate resolution and repeatability while offering much greater flexibility, enabling 2D measurements on strongly curved surfaces. Its compact and lightweight form factor—similar to the SAM—makes SCOM highly portable and well suited for on-machine metrology, positioning it as a practical complement to conventional high-precision interferometric methods.

**Table 1:** Summary of the key specifications of several mirror-surface metrology techniques^a^

Method	FI	RADSI	CGH	NOM/LTP	SAM	SCOM
1D/2D	2D	2D	2D	1D	1D/2D	2D
Measurement Type	Height	Height	Height	Slope	Slope/Curvature	Curvature
Radius of Curvature Range	>0.5 m	>1 m	>few mm	>1–5 m	>50–200 mm	>50–200 mm
Field of View	100–300 mm	100–300 mm	100–300 mm	1–2.5 mm	2-3 mm	20–30 mm
Lateral/Spatial Resolution	~35–50 µm	~35–50 µm	~35–50 µm	1000–2000 µm	100–300 µm	100–300 µm
Repeatability (rms)	0.1–2 nm	0.5–3 nm	1–5 nm	0.5–5 nm	0.5–5 nm	5–25 nm
Acquisition Time	3 h	4 h	3 h	1 h	1 h	3.5 h
Data Processing Time	0.5 h	1 h	3 h	<0.1 h	0.5 h	2 h
Portability	Low	Low	Low	Medium	High	High

^a^Acquisition and data processing times are representative estimates for a single stitched scan of a 100mm-long mirror with high optical quality and the radius of curvature range for CGH is defined by CGH

After the demonstration of this instrument of metrology for various mirrors, we plan to improve the device further by enhancing its stability, robustness and data acquisition. In addition, the components, working mode and data processing of the SCOM can be adjusted to optimize its performance as required for different operations, granting it great potential and flexibility. For instance, the present measurement speed is limited due to the significant time taken in using the linear motor for the diffuser in step-scan mode. The measurement speed can be further enhanced by applying the advanced fly scan technique^48^. This curvature metrology technique can be suitable for high-precision measurement of strongly curved and freeform mirrors, and it expands the capabilities of current metrology instruments to mirrors that currently cannot be made because they cannot be measured. In addition, the 2D curvature maps provide much rich information about the surface profile of X-ray mirrors.^49^ For instance, irregularities in the mirror curvature are directly linked to distortions in the defocused X-ray stripe, making 2D curvature maps essential for predicting and optimizing beam quality.^50,51^ It can offer unique advantages for metrology of not only X-ray mirrors for synchrotron radiation, free electron laser and astronomical and space science, but also freeform mirrors in advanced industrial products.

## Materials and methods

**Fig. 8:**
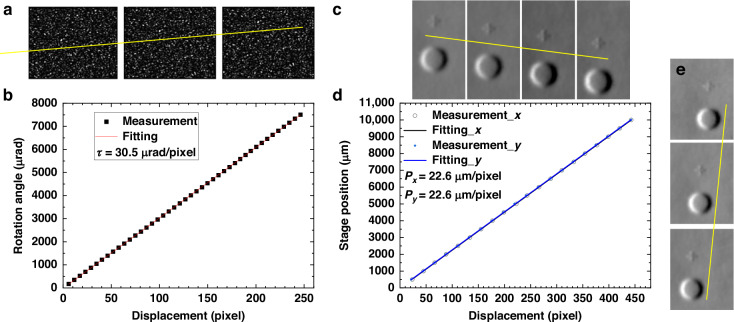
Calibration of angular scaling factor τ and effective pixel size P. **a** Speckle images collected at three different mirror angles. **b** Change in mirror rotation angle against retrieved speckle displacement from the raw speckle image. **c**, **e** Retrieved horizontal slope of the mirror by shifting it in steps of 1500 μm along the horizontal and vertical directions, respectively. **d** Stage position against retrieved displacement from the shifted slope images

To precisely integrate the slope and height maps for the mirror surface from the retrieved curvature information, it is crucial to calibrate both the angular scaling factor and spatial scaling factor. To demonstrate the calibration procedure, the SCOM mounted on the IBF system has been used here. For calibrating the angular scaling factor, the SUT was mounted on a rotating stage, and the angle was varied with a constant step size of 100 μrad, with the speckle images were recorded by the camera at each step. As shown in Fig. 8a, the speckle pattern shifts as the rotation angle changes. By applying the cross-correlation algorithm, the speckle displacement with angle can be tracked with sub-pixel accuracy. The rotation angle is plotted as a function of the retrieved speckle displacement in Fig. 8b, and the angular scaling factor can be determined from best-fit slope of τ = 38.5μrad/pixel.

Due to the scattering and diffraction of the laser by the diffuser, the speckle beam is divergent from the diffuser up to the mirror and the camera. Here, the spatial scaling factor can be calibrated using a clearly defined feature on the mirror surface, by moving the mirror in front of the SCOM and tracking the displacement of these features in the curvature or slope maps. As shown in Fig. 8c, e, the SUT was moved along both the horizontal and vertical directions with a step size of 1500 μm. The cross-correlation algorithm was used again for the retrieved slope maps in both scanning directions, and the displacement between the features on the surface—here, a circular BRF and a cross—can be calculated. Figure 8d shows the tracked displacement as a function of the subset window of constant offset, and it confirms that the speckle beam distributes equally on the detector with the effective pixel size $P=22.6{\rm{\mu }}{\rm{m}}/{\rm{pixel}}$. This yields the corresponding spatial scaling factor ${\rm{\varsigma }}=0.50$ with the physical pixel size $p=45.1{\rm{\mu }}{\rm{m}}/{\rm{pixel}}$ (after binning).

**Fig. 9:**
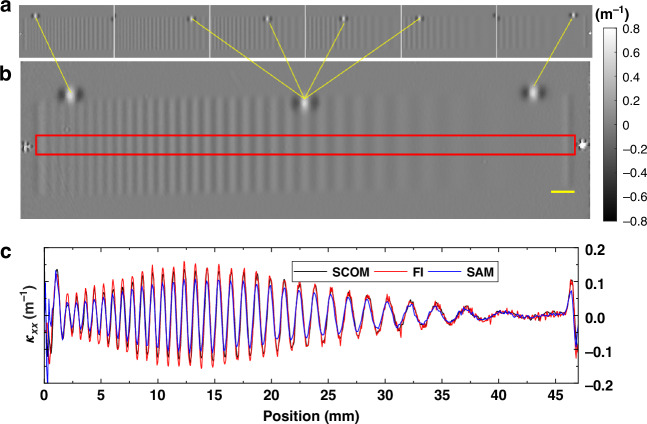
Demonstration of stitching curvature images. **a** Multiple retrieved curvature images with stage translation step of 5 mm. **b** Stitched curvature map generated from the individual images in (**a**). **c** Line profile extracted from the stitched curvature map shown in (**b**) and compared with the one measured by FI and SAM. The scale bar is 2 mm

Figure 9 demonstrates the capability of the SCOM system to perform curvature map stitching, enabling high-resolution measurements over large optical surfaces beyond a single field of view. Figure 9a shows a series of retrieved curvature images acquired by translating the mirror in 5 mm steps using the motorized stage. Each image represents the local curvature measured within the limited field of view of the SCOM, with partial overlap between adjacent scans to ensure alignment and continuity. These individual curvature maps are then combined through a stitching algorithm that aligns overlapping regions and corrects for any angular misalignment between measurements.^52^ The result of this stitching process is presented in Fig. 9b, which displays a continuous and seamless curvature map over a larger measurement area. A spatially varying chirp with constant height amplitude of 5 nm and spatial period from 10 mm down to 0.75 mm was created on the surface with IBF using an 0.75 mm × 8 mm rectangular aperture. The stitched map preserves both low- and high-frequency curvature features, demonstrating the spatial consistency and accuracy of the SCOM system across multiple scans.

To further illustrate the validity of the stitched data, a line profile is extracted from the stitched curvature map and compared the one from FI and SAM shown in Fig. 9c. Overall, the stitched profile from SCOM agrees well with the one from FI and SAM for larger period and shows smooth transitions across boundaries and accurately captures the surface curvature variation of the chirp pattern, validating the robustness of the stitching algorithm. The decreasing amplitude at shorter spatial periods is expected, due to reduced etching efficiency as the period approaches the size of the removal function. It should also be noted that the measured amplitude of the chirp curvature at short periods is larger for FI than for SCOM. This is due to the higher spatial resolution of the FI (around 46 µm) compared to the SCOM, which has an estimated resolution of approximately 200 μm based on its effective pixel size and processing window (w *=* 11). This demonstration confirms that the SCOM system can be effectively used for large-aperture optics by acquiring and combining multiple overlapping measurements. The stitching approach not only extends the measurement range of the system but also maintains moderate spatial resolution and precision, making it suitable for full-aperture characterization of freeform or segmented mirror systems.

The core of the technique involves tracking the shift of each pixel between two speckle image stacks. A small window (typically 5 ~ 11 pixels) centred on each pixel in the first image stack is compared to a slightly larger window (with ~5-pixel margin) in the second image stack using Pearson correlation coefficients.^53^ The peak of the correlation map indicates the displacement, refined to sub-pixel accuracy using polynomial fitting. Given the computational intensity of performing 2D correlation on high-resolution images (e.g., 1152 × 886), the algorithm is optimized for parallel execution on multicore CPUs or GPUs. Using a Dell Precision 5820 workstation (256 GB RAM, Quadro RTX 5000 GPU), the processing time for this dataset with a window size of 11, a margin of 5, and 51 frames is approximately 35 s on a GPU and 4505 s on a CPU. To obtain the slope and height maps from the curvature maps, integration was performed using the two-dimensional discrete cosine transform method,^44^ which provides very fast computation speed and excellent performance without the need to set additional parameters.

## Supplementary information

Supplementary Information for Speckle-based curvature optical metrology

## Data Availability

All data are available in the main text or the supplementary materials. Additional data supporting the findings of this study are available from the corresponding author upon reasonable request.

## References

[1] Salditt, T. & Osterhoff, M. X-ray focusing and optics. in Nanoscale Photonic Imaging (eds Salditt, T., Egner, A. & Luke, D. R.) (Cham: Springer, 2000), 71-124.

[2] Mimura, H. et al. Breaking the 10 nm barrier in hard-X-ray focusing. *Nat. Phys.* **6**, 122–125 (2010).

[3] Morawe, C. et al. Differential deposition for figure correction of x-ray mirrors. Proceedings of SPIE 11108, Advances in X-Ray/EUV Optics and Components XIV. San Diego, CA, USA: SPIE, 2019, 1110807.

[4] Shurvinton, R. et al. Ion beam figuring for X-ray mirrors: history, state-of-the-art and future prospects. *J. Synchrotron Radiat.* **31**, 655–669 (2024). 38771776 10.1107/S1600577524002935 PMC11226173

[5] Yamauchi, K. et al. Figuring with subnanometer-level accuracy by numerically controlled elastic emission machining. *Rev. Sci. Instrum.* **73**, 4028–4033 (2002).

[6] Ke, X. L. et al. Review on robot-assisted polishing: status and future trends. *Robot. Computer-Integr. Manuf.* **80**, 102482 (2023).

[7] Thiess, H., Lasser, H. & Siewert, F. Fabrication of X-ray mirrors for synchrotron applications. *Nucl. Instrum. Methods Phys. Res. Sect. A: Accelerators, Spectrometers, Detect. Associated Equip.* **616**, 157–161 (2010).

[8] Qian, S. N. & Takacs, P. Nano-accuracy surface figure metrology of precision optics. in Modern Metrology Concerns (ed Cocco, L.) (Rijeka: InTech, 2012), 77-114.

[9] Rommeveaux, A. V., Lantelme, B. & Barrett, R. ESRF metrology laboratory: overview of instrumentation, measurement techniques, and data analysis. Proceedings of SPIE 7801, Advances in Metrology for X-Ray and EUV Optics III. San Diego, CA, USA: SPIE, 2010, 780107.

[10] Yashchuk, V. V. et al. Super-resolution surface slope metrology of x-ray mirrors. *Rev. Sci. Instrum.* **91**, 075113 (2020). 32752867 10.1063/5.0005556

[11] Alcock, S. G., Nistea, I. & Sawhney, K. Nano-metrology: the art of measuring X-ray mirrors with slope errors <100 nrad. *Rev. Sci. Instrum.* **87**, 051902 (2016). 27250374 10.1063/1.4949272

[12] Lee, C. S. et al. Dual graphene films growth process based on plasma-assisted chemical vapor deposition. Proceedings of SPIE 7761, Carbon Nanotubes, Graphene, and Associated Devices III. San Diego, CA, USA: SPIE, 2010, 77610P.

[13] de Groot, P. Principles of interference microscopy for the measurement of surface topography. *Adv. Opt. Photonics* **7**, 1–65 (2015).

[14] García-Moreno, A., Belenguer-Dávila, T. & González-Fernández, L. M. Freeform mirror validation by interferometric techniques using a spatial light modulator. *Opt. Contin.* **2**, 1605–1615 (2023).

[15] Scheiding, S. et al. Freeform mirror fabrication and metrology using a high performance test CGH and advanced alignment features. Proceedings of SPIE 8613, Advanced Fabrication Technologies for Micro/Nano Optics and Photonics VI. San Francisco, CA, USA: SPIE, 2013, 86130J.

[16] Poleshchuk, A. G., Nasyrov, R. K. & Asfour, J. M. Combined computer-generated hologram for testing steep aspheric surfaces. *Opt. Express* **17**, 5420–5425 (2009). 19333307 10.1364/oe.17.005420

[17] Vivo, A. et al. Stitching methods at the European Synchrotron Radiation Facility (ESRF). *Rev. Sci. Instrum.* **87**, 051908 (2016). 27250380 10.1063/1.4950745

[18] Huang, L. et al. Two-dimensional stitching interferometry based on tilt measurement. *Opt. Express* **26**, 23278–23286 (2018). 30184981 10.1364/OE.26.023278

[19] Polack, F. et al. Surface shape determination with a stitching Michelson interferometer and accuracy evaluation. *Rev. Sci. Instrum.* **90**, 021708 (2019). 30831756 10.1063/1.5061930

[20] da Silva, M. B. et al. A Fizeau interferometry stitching system to characterize X-ray mirrors with sub-nanometre errors. *Opt. Lasers Eng.* **161**, 107192 (2023).

[21] Huang, L. et al. Two-dimensional stitching interferometry for self-calibration of high-order additive systematic errors. *Opt. Express* **27**, 26940–26956 (2019). 31674564 10.1364/OE.27.026940

[22] Nicolas, J. et al. Completeness condition for unambiguous profile reconstruction by sub-aperture stitching. *Opt. Express* **26**, 27212–27220 (2018). 30469794 10.1364/OE.26.027212

[23] Mimura, H. et al. Relative angle determinable stitching interferometry for hard x-ray reflective optics. *Rev. Sci. Instrum.* **76**, 045102 (2005).

[24] Javidi, B. et al. Roadmap on digital holography [Invited]. *Opt. Express* **29**, 35078–35118 (2021). 34808951 10.1364/OE.435915

[25] Micó, V. et al. Resolution enhancement in quantitative phase microscopy. *Adv. Opt. Photonics* **11**, 135–214 (2019).

[26] Zeng, T. J., Zhu, Y. M. & Lam, E. Y. Deep learning for digital holography: a review. *Opt. Express* **29**, 40572–40593 (2021). 34809394 10.1364/OE.443367

[27] Wen, K. et al. Spherical wave illumination scanning digital holographic profilometry. *Opt. Express* **32**, 1609–1624 (2024). 38297709 10.1364/OE.507233

[28] Alcock, S. G. et al. The Diamond-NOM: a non-contact profiler capable of characterizing optical figure error with sub-nanometre repeatability. *Nucl. Instrum. Methods Phys. Res. Sect. A: Accelerators, Spectrometers, Detect. Associated Equip.* **616**, 224–228 (2010).

[29] Siewert, F. et al. The nanometer optical component measuring machine: a new sub-nm topography measuring device for X-ray optics at BESSY. *AIP Conf. Proc.* **705**, 847–850 (2004).

[30] Qian, S. N., Jark, W. & Takacs, P. Z. The Penta-prism LTP: a long-trace-profiler with stationary optical head and moving Penta prism. *Rev. Sci. Instrum.* **66**, 2562–2569 (1995).

[31] Takacs, P. Z., Qian, S. N. & Colbert, J. Design of a long trace surface profiler. Proceedings of SPIE 0749, Metrology: Figure and Finish. Los Angeles, CA, USA: SPIE, 1987, 59-64.

[32] Yashchuk, V. V. et al. Sub-microradian surface slope metrology with the ALS Developmental Long Trace Profiler. *Nucl. Instrum. Methods Phys. Res. Sect. A: Accelerators, Spectrometers, Detect. Associated Equip.* **616**, 212–223 (2010).

[33] Wang, H. C., Moriconi, S. & Sawhney, K. Nano-precision metrology of X-ray mirrors with laser speckle angular measurement. *Light Sci. Appl.* **10**, 195 (2021). 34552044 10.1038/s41377-021-00632-4 PMC8458457

[34] Gao, W. et al. On-machine and in-process surface metrology for precision manufacturing. *CIRP Ann.* **68**, 843–866 (2019).

[35] Majhi, A. et al. Sub-nanometre quality X-ray mirrors created using ion beam figuring. *J. Synchrotron Radiat.* **31**, 706–715 (2024). 38904938 10.1107/S1600577524004594 PMC11226171

[36] Pradhan, P. et al. Ultra-high precision NiP mirror fabrication using ion beam figuring for space applications. *Opt. Express* **33**, 17721–17734 (2025). 40798002 10.1364/OE.549555

[37] Berujon, S. & Ziegler, E. Near-field speckle-scanning-based x-ray tomography. *Phys. Rev. A* **95**, 063822 (2017).

[38] Wang, H. C. & Sawhney, K. Hard X-ray omnidirectional differential phase and dark-field imaging. *Proc. Natl. Acad. Sci. USA* **118**, e2022319118 (2021). 33619105 10.1073/pnas.2022319118 PMC7936267

[39] Wang, H. C., Kashyap, Y. & Sawhney, K. From synchrotron radiation to lab source: advanced speckle-based X-ray imaging using abrasive paper. *Sci. Rep.* **6**, 20476 (2016). 26847921 10.1038/srep20476 PMC4742822

[40] Wang, H. C., Kashyap, Y. & Sawhney, K. Hard-X-ray directional dark-field imaging using the speckle scanning technique. *Phys. Rev. Lett.* **114**, 103901 (2015). 25815933 10.1103/PhysRevLett.114.103901

[41] Pan, B. et al. Performance of sub-pixel registration algorithms in digital image correlation. *Meas. Sci. Technol.* **17**, 1615–1621 (2006).

[42] Wang, H. C., Sutter, J. & Sawhney, K. Advanced in situ metrology for x-ray beam shaping with super precision. *Opt. Express* **23**, 1605–1614 (2015). 25835918 10.1364/OE.23.001605

[43] Kottler, C. et al. A two-directional approach for grating based differential phase contrast imaging using hard x-rays. *Opt. Express* **15**, 1175–1181 (2007). 19532346 10.1364/oe.15.001175

[44] Huang, L. et al. Comparison of two-dimensional integration methods for shape reconstruction from gradient data. *Opt. Lasers Eng.* **64**, 1–11 (2015).

[45] Morawe, C. et al. Graded multilayers for figured Kirkpatrick-Baez mirrors on the new ESRF end station ID16A. Proceedings of SPIE 9588, Advances in X-Ray/EUV Optics and Components X. San Diego, CA, USA: SPIE, 2015, 958803.

[46] Wang, H. C. et al. Development of an advanced in-line multilayer deposition system at Diamond Light Source. *J. Synchrotron Radiat.* **31**, 1050–1057 (2024). 39120915 10.1107/S1600577524006854 PMC11371021

[47] Yashchuk, V. V., Samoylova, L. V. & Kozhevnikov, I. V. Specification of x-ray mirrors in terms of system performance: new twist to an old plot. *Optical Eng.* **54**, 025108 (2015).

[48] Wang, H. C. et al. High-energy, high-resolution, fly-scan X-ray phase tomography. *Sci. Rep.* **9**, 8913 (2019). 31222085 10.1038/s41598-019-45561-w PMC6586786

[49] Roddier, F. Curvature sensing and compensation: a new concept in adaptive optics. *Appl. Opt.* **27**, 1223–1225 (1988). 20531543 10.1364/AO.27.001223

[50] Hu, L. F. et al. Investigation of the stripe patterns from X-ray reflection optics. *Opt. Express* **29**, 4270–4286 (2021). 33771010 10.1364/OE.417030

[51] Hu, L. F. et al. Research on the beam structures observed from X-ray optics in the far field. *Opt. Express* **31**, 41000–41013 (2023). 38087509 10.1364/OE.499685

[52] Preibisch, S., Saalfeld, S. & Tomancak, P. Globally optimal stitching of tiled 3D microscopic image acquisitions. *Bioinformatics* **25**, 1463–1465 (2009). 19346324 10.1093/bioinformatics/btp184 PMC2682522

[53] Vo. N. T. et al. Practical implementations of speckle-based phase-retrieval methods in Python and GPU for tomography. Proceedings of SPIE 12242, Developments in X-Ray Tomography XIV. San Diego, CA, USA: SPIE, 2022, 122420E

