# Supplementary material for: Speckle-based curvature optical metrology
Source: Light Sci Appl. 2026 Apr 8;15:192. doi: 10.1038/s41377-026-02257-x (PMC13062094; doi:10.1038/s41377-026-02257-x)
Supplement: Supplementary file 1 — Supplementary Information for Speckle-based curvature optical metrology [file 41377_2026_2257_MOESM1_ESM.docx]

Supplementary Information for

1. **Speckle-based curvature optical metrology**

Hongchang Wang^1^*, Riley Shurvinton, Paresh Pradhan and Kawal Sawhney

Diamond Light Source Ltd, Harwell Science and Innovation Campus, Didcot, OX11 0DE, UK

1. *email: hongchang.wang@diamond.ac.uk

**SI S1. Retrieved curvature maps for IBF process**


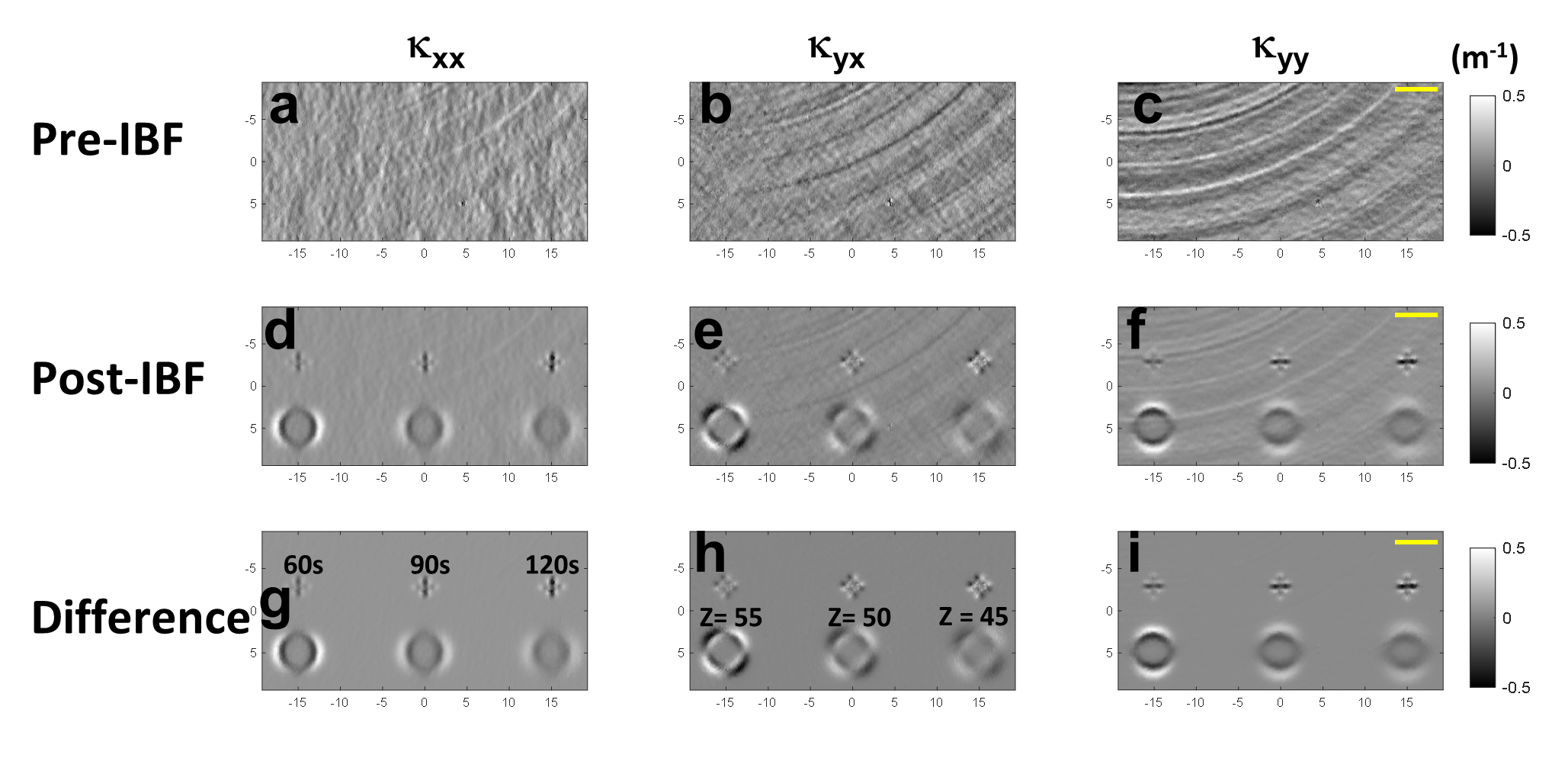


**Figure S1 | Retrieved curvature maps for a test mirror before and after ion beam etching.** (a–c): Retrieved horizontal, diagonal, and vertical curvature maps of the test mirror before etching. (d–f): Corresponding curvature maps after etching. (g–i): Curvature change maps (horizontal, diagonal, and vertical), representing the etched profile obtained by subtracting the pre-etching from the post-etching maps. The scale bar (horizontal yellow solid lines) is 5mm.

**Figure S1 (a–c)** show the curvature distributions along the horizontal, diagonal, and vertical directions, respectively, prior to etching. After etching, the mirror was remeasured and the corresponding post-etching curvature maps are shown in **Figure S1 (d–f)**, where three circular BRF and three cross marks can be observed. To visualize the BRF profile, **Figure S1 (g–i)** present the curvature change maps in the same three directions, obtained by subtracting the pre-IBF maps from the post-IBF maps. The tooling marks from the substrate have been fully removed and only the etched profiles are shown. By retrieving absolute curvature maps after each etching iteration, this approach is referred to as the absolute mode.

**SI S2. Repeatability improvement**


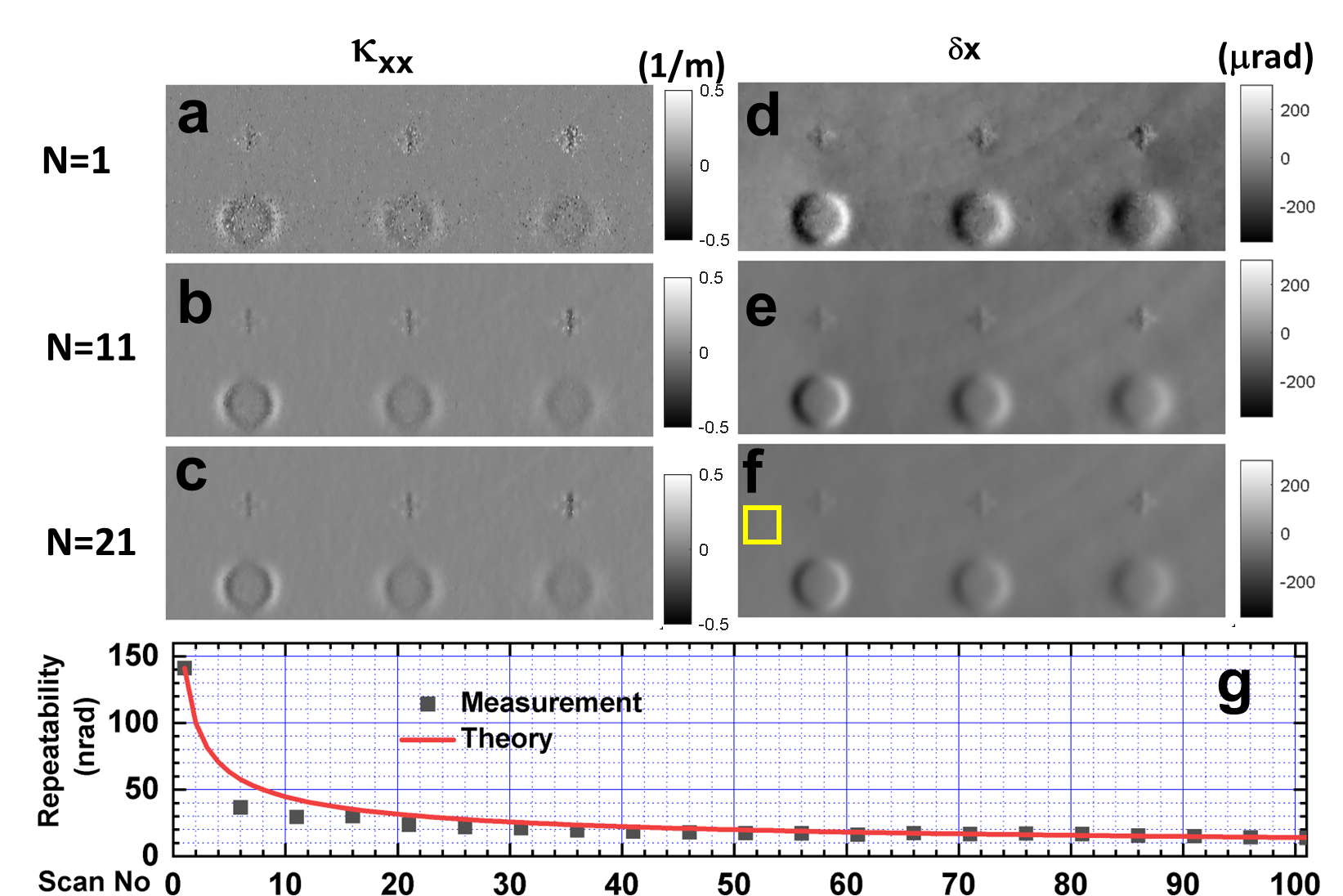


**Figure S2 | Validation of the repeatability of the SCOM system using retrieved curvature and slope maps with varying numbers of speckle images.** (a–c): Retrieved curvature maps (d–f): Retrieved slope maps using 1, 11, and 21 speckle images, respectively. (g): Repeatability of the SCOM system installed on the IBF platform as a function of the number of speckle scans.

**Figure S2** presents the validation of the repeatability performance of the SCOM system by analyzing the impact of the number of speckle images used in the slope retrieval process. **Figure S2 (a–f)** show the retrieved horizontal curvature and slope maps of a test mirror when using 1, 11, and 21 speckle images, respectively. As expected, the slope map obtained from a single speckle image **Figure S2 (a)** and **(d)** shows noticeable noise and small-scale artifacts due to speckle grain randomness and camera noise. When the number of speckle images is increased to 11 **Figure S2 (b)** and **(e)** , the curvature and slope map becomes smoother and more stable, indicating a reduction in random error. With 21 images **Figure S2 (c)** and **(f),** the curvature and slope map exhibits more uniformity and repeatability, demonstrating the effectiveness of image averaging in suppressing noise and enhancing measurement stability. To quantify this behaviour, **Figure S2 (g)** shows the repeatability of the SCOM system—defined as the standard deviation of the slope differences between individual scans and the average slope within a selected square region on the mirror surface—as a function of the number of speckle scans. The results reveal a clear trend: increasing the number of speckle images used for averaging significantly improves measurement repeatability. The improvement follows an expected noise-reduction curve, where noise decreases approximately proportionally to the square root of the number of images. This trend confirms that the primary source of variation is random noise, and that averaging effectively mitigates its impact. These findings validate the high repeatability and reliability of the SCOM system, even when integrated into the IBF platform, where environmental disturbances such as vibration and thermal drift could potentially impact measurement performance. By optimizing the number of speckle images, users can achieve a balance between measurement time and precision, making the system adaptable for both fast coarse measurements and slow high-precision evaluations.

**SI S3. Temperature, Humidity and Stability of SCOM System**


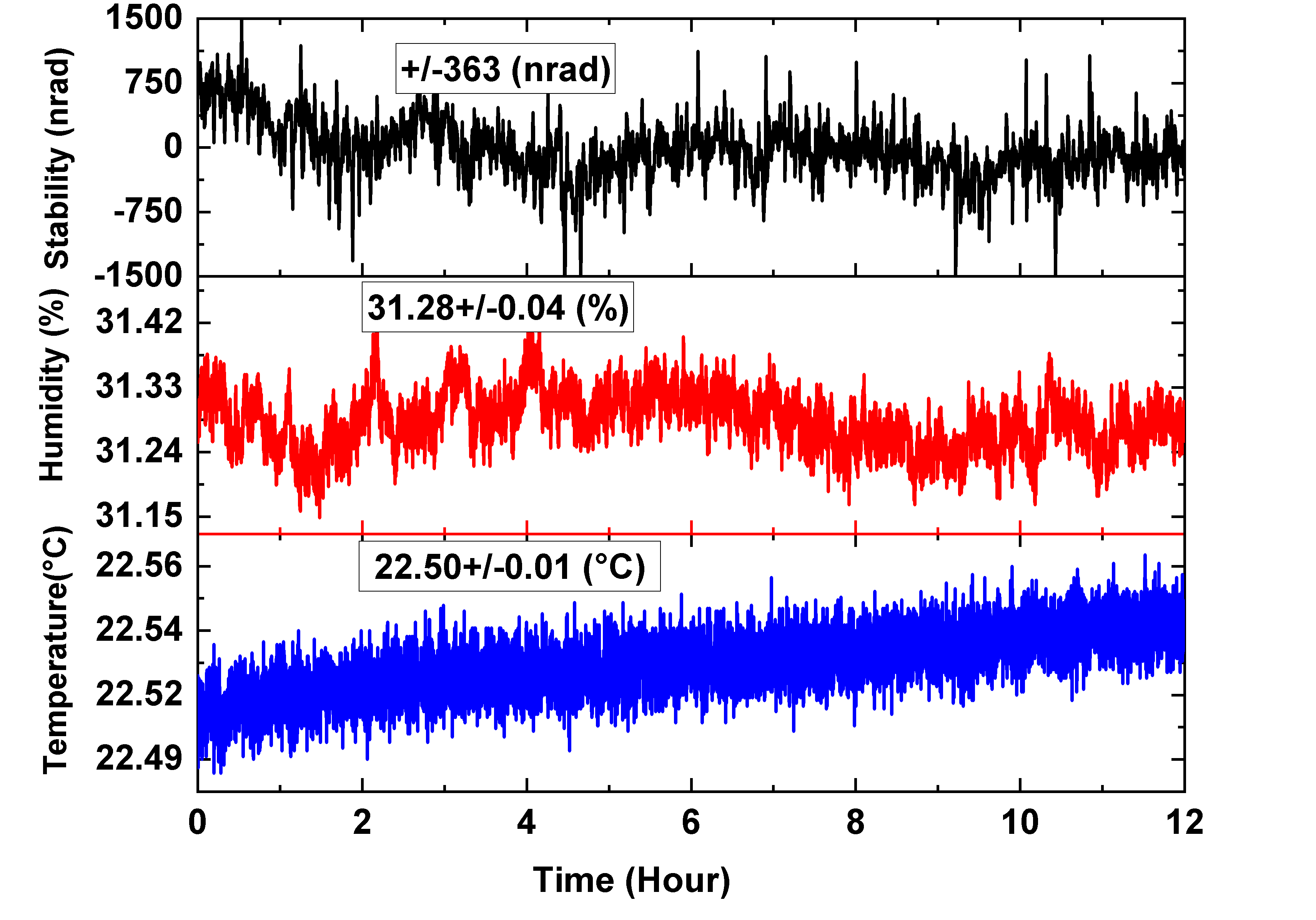


**Figure S3. | The SCOM enclosure temperature, humidity of the metrology lab and the stability measurement for SCOM over 12 hours.**

To ensure stable measurement conditions, one SCOM system is housed within a thermally isolated enclosure. The temperature inside the enclosure is not actively regulated; instead, stability is achieved through the large heat capacity of the granite bench and the minimal power dissipation of the internal components. To characterize the intrinsic noise level of the SCOM system, we performed a stability scan with the SCOM optical head held fixed. As shown in **Figure S3**, the temperature in the optics metrology room exhibits a standard deviation of only 0.01 °C over 12 hours, while the relative humidity varies by just 0.04%. This high level of thermal stability also suppresses air currents, thereby improving the stability of the speckle pattern. Under these conditions, the system achieved a stability of 363 nrad.

**SI S4. Simulation of integration error from the measured noise from curvature**


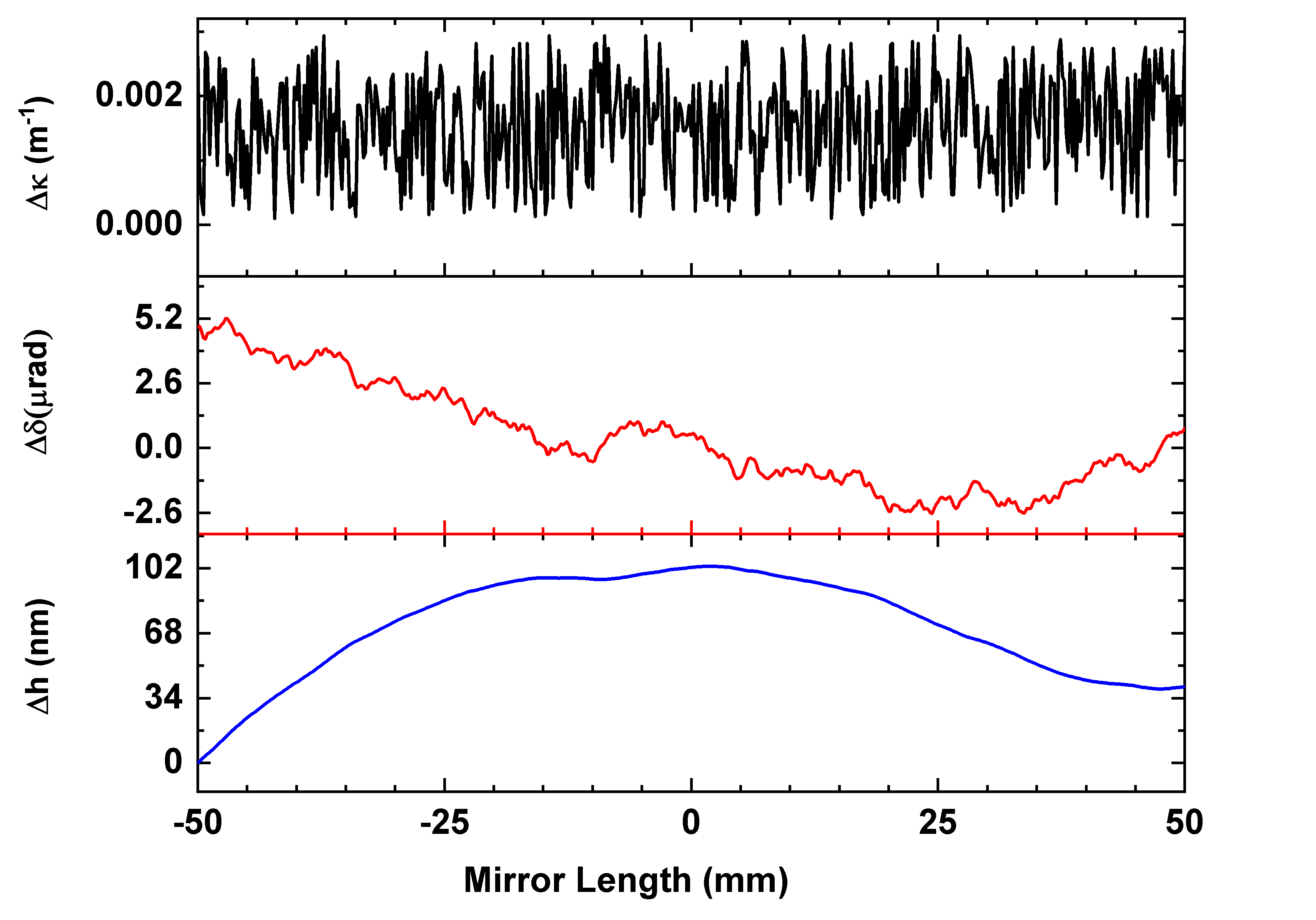

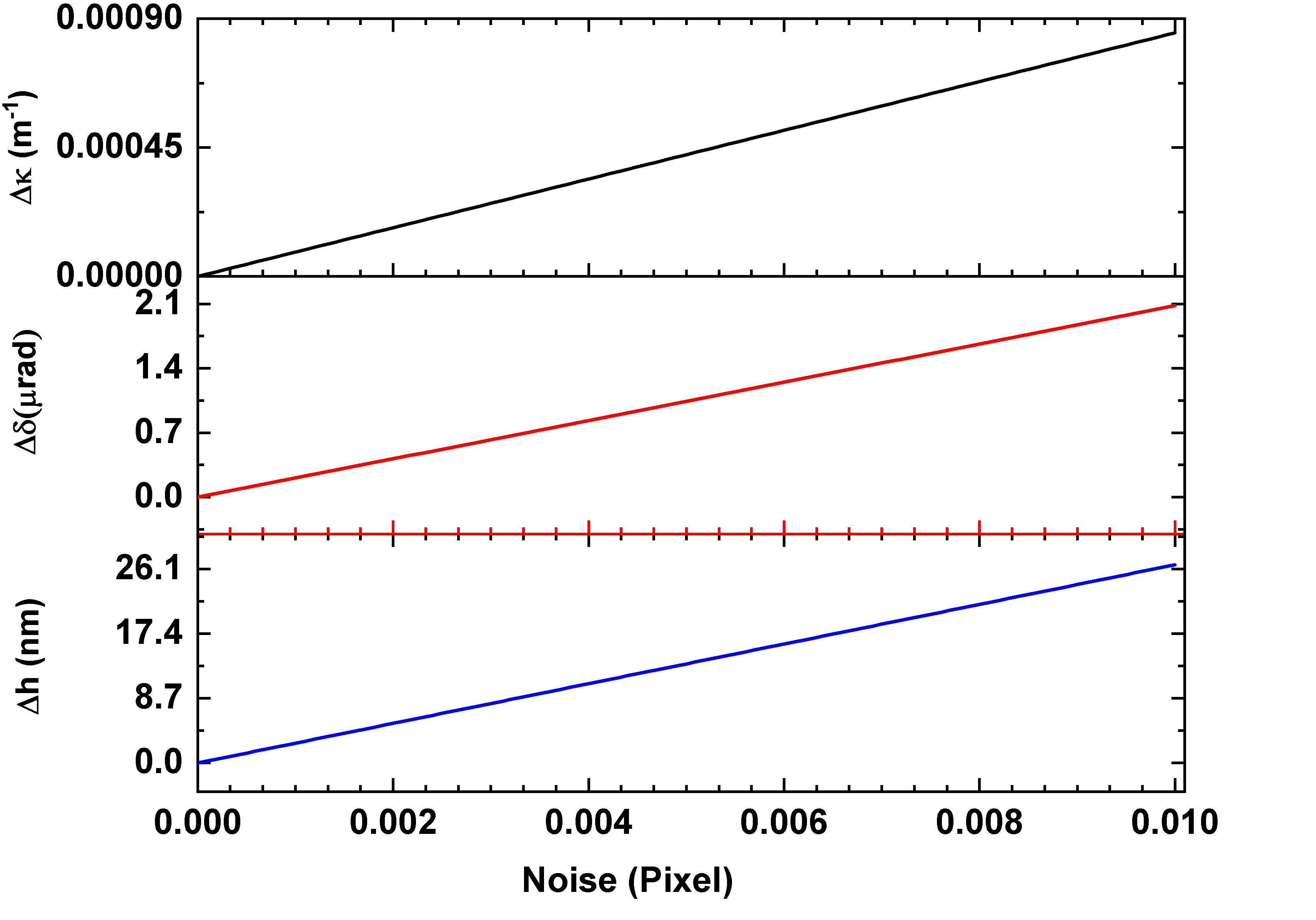


**Figure S4. | The simulation of the two integrations to slope and height from the curvature. (Left) the simulated curvature error, and integrated slope and height error with SCOM instrument noise of 0.01 pixel with ASF=56.7urad pixel^-1^. (Right), the simulated curvature error, and integrated slope and height error with SCOM instrument as function of SCOM instrument noise.**

The simulation of the retrieved curvature, slope, and height errors was performed by adding tracking-displacement noise (in pixels) for a 100-mm-long mirror measurement. The ASF was set to 56.7 urad pixel^-1^, consistent with the standard SCOM configuration. As shown in **Figure S4 (left)**, the standard deviation of the retrieved curvature is 0.0009 m⁻¹ for a tracking error of 0.01 pixel, while the corresponding integrated slope and height errors are 2.0 µrad rms and 25 nm rms, respectively. **Figure S4 (right)** shows that the standard deviations of curvature, slope, and height error increase proportionally with the noise level from 0 to 0.01 pixel. To minimize tracking noise, it is essential to reduce all sources of random error, including improving the stability of the SCOM system and providing a better metrology environment to suppress air turbulence.

**SI S5. Error budget of SCOM system**


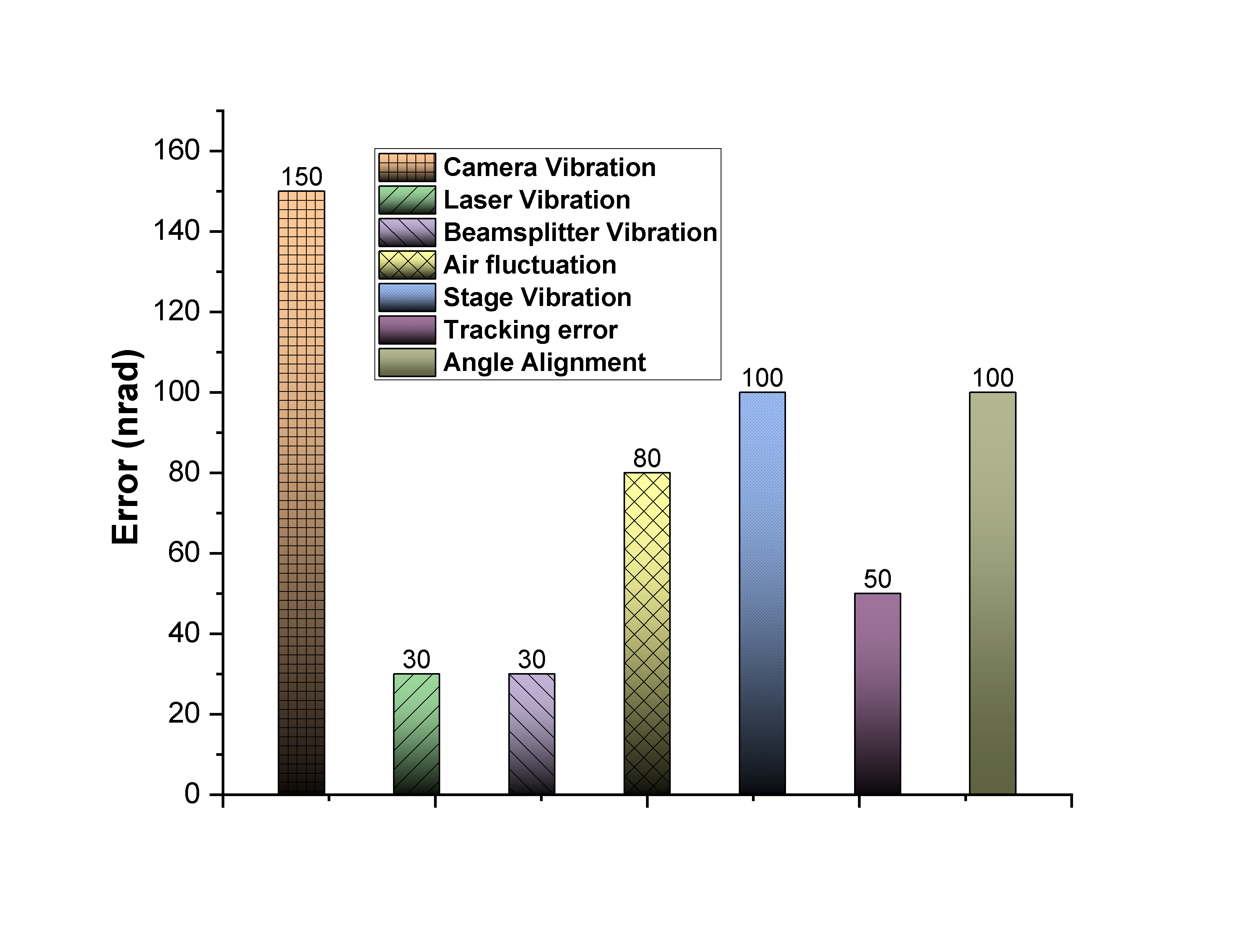


**Figure S5. | The estimated error budget of SCOM system.**

To quantify the error budget of the SCOM system, both random and systematic error sources were considered. The random errors include camera vibration, laser and beamsplitter vibration, air fluctuations, stage vibration, and speckle-tracking noise. The primary systematic contribution arises from angular misalignment. As shown in **Figure S5** for the SCOM system in an enclosure with the test mirror on Hexapod stage, the total random-error magnitude of the SCOM system is approximately 210 nrad, while the systematic error caused by angular misalignment is on the order of 100 nrad.

**SI S6. Spatial Resolution**


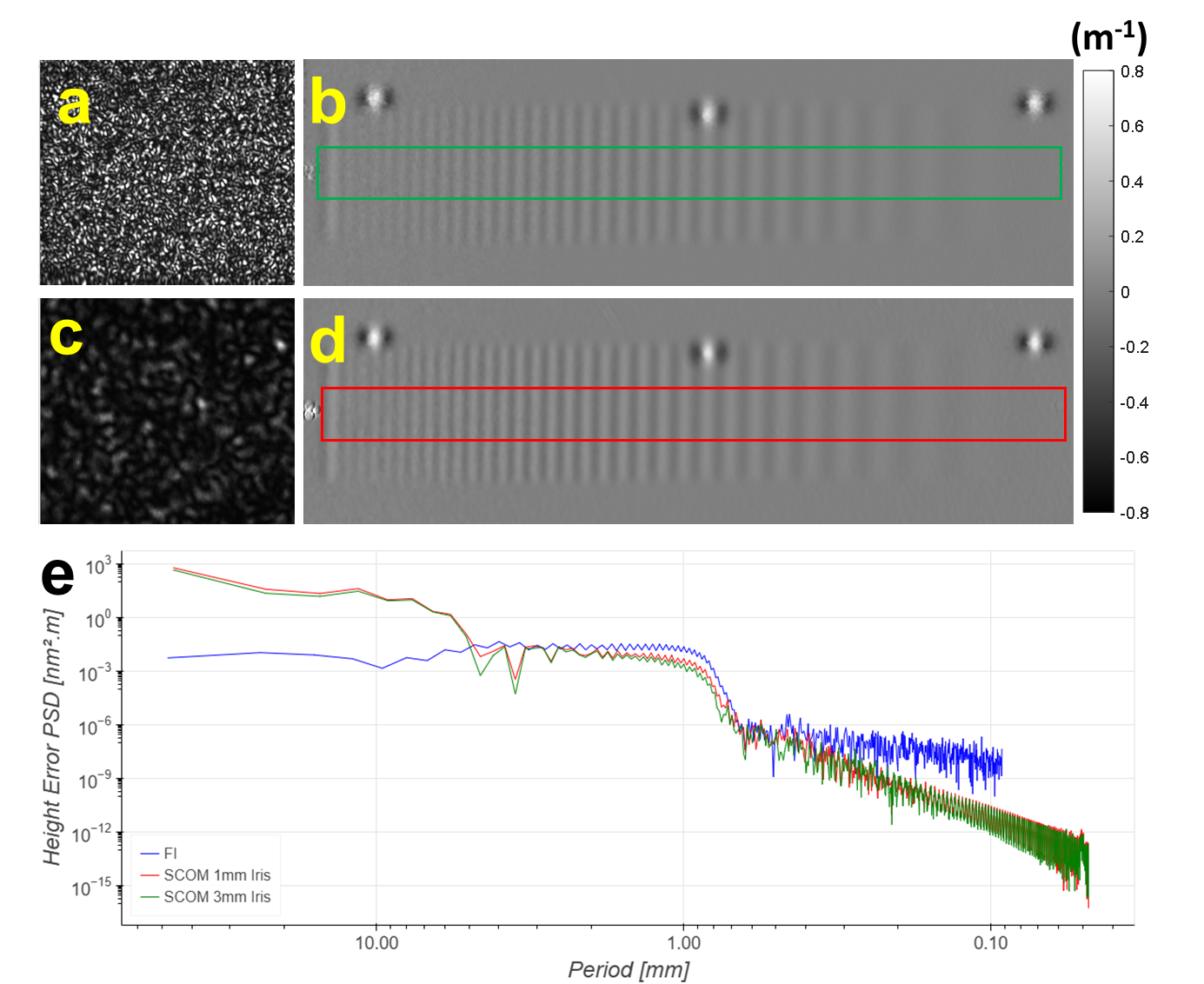


**Figure S6. | SCOM measurement of a chirp mirror with different iris size. (a, b): Speckle image and measured curvature R_XX_ with iris size of 3mm, (c, d): Speckle image and measured curvature R_XX_ with iris size of 1mm; (e): PSD curve for the selected region compared to the data measured with FI.**

To evaluate the spatial resolution of the SCOM, a mirror with a chirped profile was measured using different iris sizes. As shown in **Figure S6 (a)**, the speckle size decreases when a larger iris is used; however, the corresponding retrieved horizontal curvature map shows reduced visibility of the small chirp features. Conversely, reducing the iris diameter from 3 mm to 1 mm increases the speckle size, and the chirp profile becomes noticeably more visible for the 1 mm iris compared with the 3 mm case. To quantitatively assess the spatial resolution of the SCOM system, the retrieved line profiles (indicated by the green and red rectangles in **Figures S6 (b) and S6 (d**)) were converted into height-error Power Spectral Densities (PSD) and compared with those measured using FI. The PSD plot showing a comparison between measured data at 1mm and 3mm iris diameter, which demonstrates that the larger iris size leads to lower sensitivity to shorter periods. The results indicate that while SCOM resolves the chirped structure, FI achieves better spatial resolution.

**SI S7. Scanning Step Size**


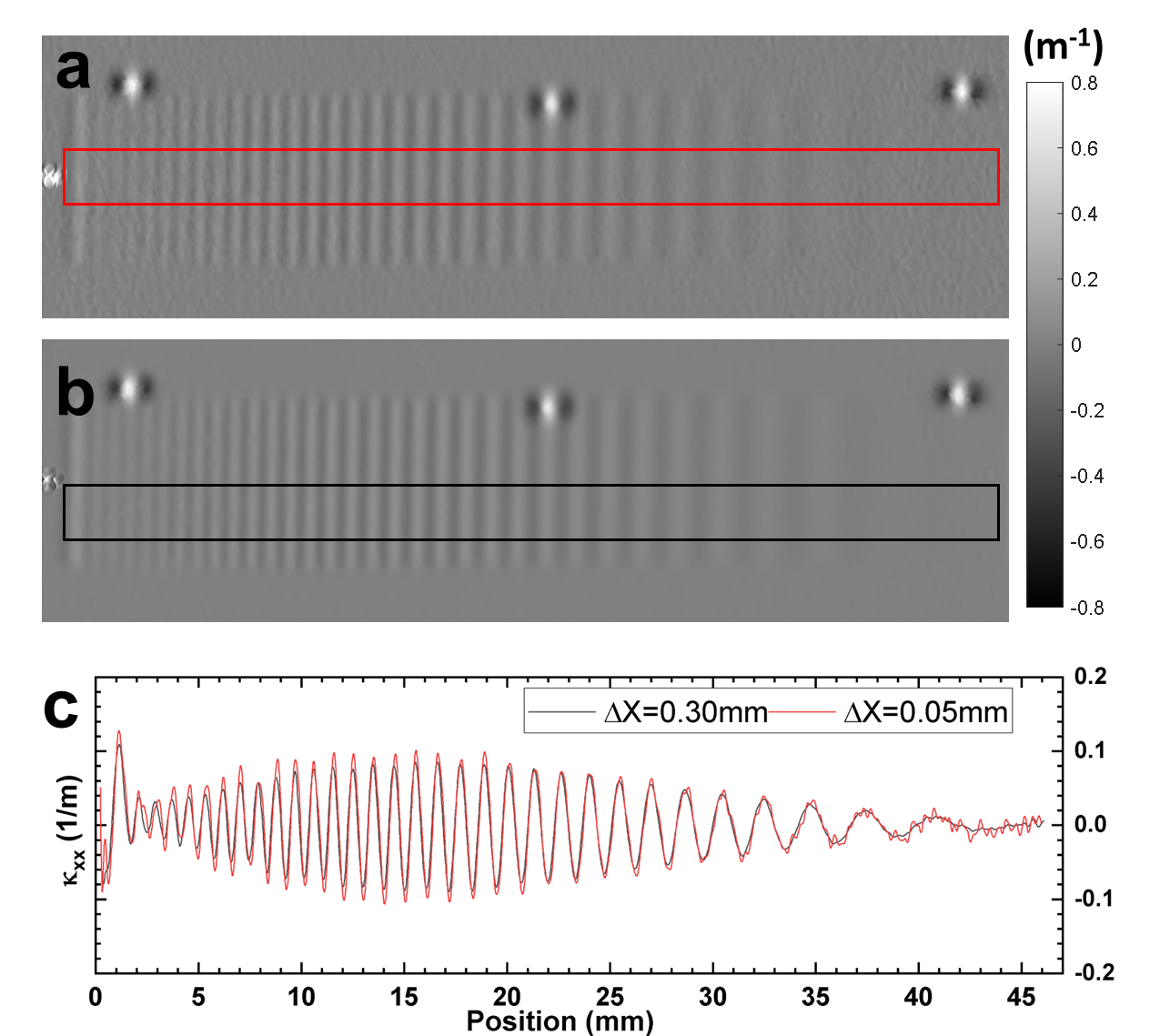


**Figure S7. | SCOM measurement of a chirp mirror with different step size. (a): Measured curvature R_XX_ with step size of 0.05mm; (b): Measured curvature R_XX_ with step size of 0.3mm; (c): Comparison of 1D line profile for both step sizes, extracted from the highlighted region in (a) and (b).**

As shown in Eq. (2), the speckle shift depends on the angular scaling factor, the mirror curvature, and the step sizes ΔX and ΔY. For a mirror with fixed curvature, smaller step sizes result in smaller speckle shifts. However, reducing the step size also increases positional uncertainty. **Figure S7 (a) and Figure S7 (b)** shows the measured curvature R_xx_ maps for step sizes ΔX = 0.3 mm and ΔX = 0.05 mm, respectively. The corresponding line profile extracted from the highlighted rectangular region is plotted in **Figure S7 (c)**. The results confirm that smaller step sizes lead to noisier data but improved spatial resolution, whereas larger step sizes yield better signal-to-noise ratios at the expense of slightly reduced spatial resolution. Therefore, selecting an appropriate step size requires balancing spatial resolution against instrument sensitivity.
